# Diffraction of a Gaussian Beam with Limited cross Section by a Volume Phase Grating under Waveguide Mode Resonance

**DOI:** 10.3390/ma14092252

**Published:** 2021-04-27

**Authors:** Volodymyr Fitio, Iryna Yaremchuk, Andriy Bendziak, Michal Marchewka, Yaroslav Bobitski

**Affiliations:** 1 Department of Photonics, Lviv Polytechnic National University, S. Bandera Str., 12, 79013 Lviv, Ukraine; volodymyr.m.fito@lpnu.ua (V.F.); iryna.y.yaremchuk@lpnu.ua (I.Y.); benzem4@gmail.com (A.B.); 2 College of Natural Sciences, Institute of Physics, University of Rzeszow, 1 Pigonia St., 35-959 Rzeszow, Poland; mmarchewka@ur.edu.pl

**Keywords:** dielectric grating, Gaussian beam, resonance, Fourier transform, sensor, sensitivity

## Abstract

In this work, the diffraction of a Gaussian beam on a volume phase grating was researched theoretically and numerically. The proposed method is based on rigorous coupled-wave analysis (RCWA) and Fourier transform. The Gaussian beam is decomposed into plane waves using the Fourier transform. The number of plane waves is determined using the sampling theorem. The complex reflected and transmitted amplitudes are calculated for each RCWA plane wave. The distribution of the fields along the grating for the reflected and transmitted waves is determined using inverse Fourier transform. The powers of the reflected and transmitted waves are determined based on these distributions. Our method shows that the energy conservation law is satisfied for the phase grating. That is, the power of the incident Gaussian beam is equal to the sum of the powers of the reflected and transmitted beams. It is demonstration of our approach correctness. The numerous studies have shown that the spatial shapes of the reflected and transmitted beams differ from the Gaussian beam under resonance. In additional, the waveguide mode appears also in the grating. The spatial forms of the reflected and transmitted beams are Gaussian in the absence of resonance. It was found that the width of the resonance curves is wider for the Gaussian beam than for the plane wave. However, the spectral and angular sensitivities are the same as for the plane wave. The resonant wavelengths are slightly different for the plane wave and the Gaussian beam. Numerical calculations for four refractive index modulation coefficients of the grating medium were carried out by the proposed method. The widths of the resonance curves decrease with the increasing in the refractive index modulation. Moreover, the reflection coefficient also increases.

## 1. Introduction

Recently, sensors for measuring the change in the refractive index, mainly of liquids, have been intensively studied. Their operation principle is based on the excitation of waveguide resonances in the grating [1,2,3,4] or in Otto–Kretschmann prismatic structures [5,6,7,8]. The resonance appears at the different wavelengths or at the different angles when the refractive index of the tested medium changes. Grating-based sensors can be of two types, namely, the dielectric grating on the dielectric substrate or metal or dielectric grating on the metal substrate [9,10,11,12]. The description and analyses of various refractive index sensors as well as providing extensive references on the corresponding sensors are presented in the reviews references [9,11]. In work [13], sensitivity of the dielectric grating on the dielectric substrate, the metal grating on the metal substrate, and the prismatic structure have been analyzed for various types of sensors. Moreover, its relationship with the waveguide properties of periodic structures has been researched. It has been shown that the ratio of the sensitivity to the width of the resonant response at full width at half maximum (FWHM) is in the range 508–522 at the resonance wavelength of 1.064 μm for surface plasmon-polariton sensors [13]. The spectral sensitivity was 76 nm in case of the dielectric grating on the dielectric substrate and the angular one was 142 mrad [10,13]. However, the widths of the resonant responses at the FWHM are very narrow. Thus, that the ratio of the sensitivity to the width of the resonant response at the FWHM is somewhat higher than 17,000. It should be noted that the dielectric gratings are recorded by the holographic method on photopolymer compositions [14,15]. The modulation amplitude of the refractive index is approximately 0.017 [16]. A low width of the resonant response is achieved due to the low modulation coefficient [17]. The highest value of the change in the refractive index is 0.48 for photopolymer compositions [18]. It corresponds to the amplitude of the change in the refractive index in a photosensitive medium of 0.24. Chalcogenide glasses are also used for recording gratings by the holographic method and the change in the refractive index can reach 0.1 in them [19]. If such dielectric grating is irradiated by a plane wave and the resonance of the waveguide modes occurs, then the reflection coefficient is equal to unity in zero diffraction order [1,2,3,17]. The calculation of the plane wave diffraction by the grating is carried out by the asymptotic RCWA [20]. However, the grating is irradiated with the limited beam over the cross section in experimental studies. It led to a significant decrease in the reflection coefficient and an expansion of the resonant response at the slight modulation of the grating medium refractive index [3,10]. The calculated RCWA resonance wavelength coincided with the experimentally measured one, though. Therefore, it is necessary to calculate the diffraction of the beam with the limited cross section. It is, for example, a Gaussian beam on such grating to predict the properties of the sensor in practical application. A relatively small number of publications are devoted to the problem of studying the diffraction of the finite diameter light beam. The study of the diffraction on the metal gratings of the finite length beam was carried out in [21]. It led to the limitation of the beam over the cross section at the excitation of surface plasmon-polariton resonances. The theoretical modeling assumes an expansion of resonances with the decrease in the grating size. This expansion is in good agreement with experimental data. In [22], diffraction of the limited beam size from 3 to 20 periods was studied using the finite-difference frequency-domain method. It was shown that with increasing beam width, the diffraction efficiency slowly converges to the values provided for by RCWA. Interesting results are presented in [23] for the reflecting gratings. Using angular spectral representation, it is shown that the fields of the Gaussian beams that are scattered by the reflecting grating differ markedly from those predicted by geometrical considerations. In [24], the effect of cross-section limited beams was studied using the rigorous boundary element method. In [25], the influence of the limited Gaussian beam on the spectrum of anomalous reflection and on the energy, distribution in the reflected beam from the waveguide with the grating was studied using an approximate theory. Lalanne and co-workers [26,27] introduced using of absorbing boundary conditions and perfect matching of layers at the ends of the unit cell to numerically analyze finite periodic structures. Guizal et al. [28] developed the method called aperiodic RCWA (ARCWA) in which the dielectric constant of the finite grating is represented as the Fourier integral. It leads to the integro-differential equation that can be solved using discretization in Fourier space. However, this method requires several hundred harmonics for convergence and requires significant computer resources. In [29], an attempt was made to extend the RCWA method for gratings with the finite number of periods using supercells, which resulted to a significant increase in the calculation time.

A simple theory of the reflection of the beam with limited cross section on the grating in which the resonance of waveguide modes is occurred, given in [30]. It provides using of direct and inverted Fourier transforms. However, this theory uses a number of approximations, which can lead to an error in the calculations. This theory does not imply a search for the spatial distribution of the amplitude and, accordingly, the power of the wave transmitted through the grating. Thus, it is impossible to verify that the law of conservation of energy is fulfilled during diffraction by purely phase gratings, which is a criterion for the correctness of the analysis.

The approach of expanding of the cross section limited beam, for example, a Gaussian beam, is effectively used in other applications. In particular, it is to study the reflection and transmission from the Fizeau wedge [31,32,33]. Analysis of transmission of a finite-diameter Gaussian beam by the Fizeau interferential wedge is presented in these works. The fringe calculation is based on angular spectrum expansion of the complex amplitude of the incident wave field. The developed approach is applicable to any beam diameter and wedge thickness at any distance from the wedge and yields as a boundary case the fringes at plane-wave illumination.

Therefore, the development of the method for analysis of the limited cross-section beam diffraction by the grating, which would be based on the well-proven numerical method, for example, RCWA, is quite important. It is especially for periodic structures in which waveguide resonances occur at low modulation of the refractive index. After all, the numerical RCWA is asymptotically accurate [34] and it quickly converges for dielectric gratings even when the resonance of the waveguide modes occurs. The sum of the diffraction efficiencies of all orders (reflected and transmitted) calculated by RCWA corresponds to the energy conservation law for phase gratings [35]; that is, this sum should be equal to unity. The equality of the sum of unity for phase gratings can be a certain criterion for the correctness of the theory and the corresponding numerical calculations. Thus, one can hope for the creation of the effective numerical method for analyzing the diffraction of light beams with limited cross section by gratings in which waveguide resonances are realized using the Fourier transform and RCWA. In addition, it is important to determine the characteristics of sensors based on dielectric gratings for the certain range of variation in the modulation amplitude of the grating refractive index.

## 2. Theoretical Background of Method

**Figure 1:**
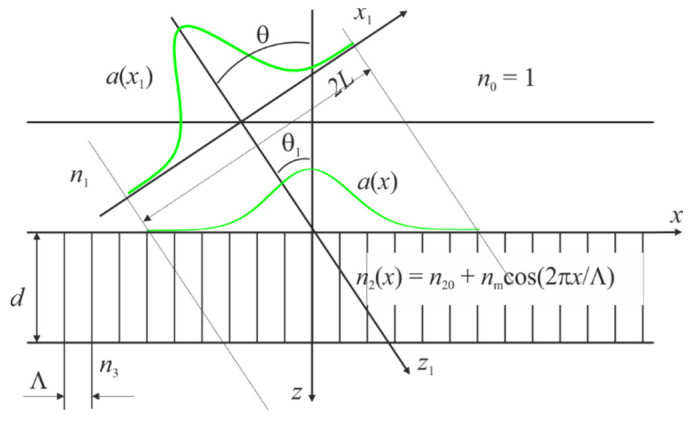
Incident of Gauss beam on the grating. The reflection coefficient is equal to 1 if the plane wave incidents on the grating and waveguide resonance occurs.

In our analysis, we consider a one-dimensional case when the wave amplitude changes along the x coordinate and does not depend on the y coordinate (see Figure 1). The limited cross-section beam is distributed in air at the angle of incidence θ to the plane of the infinite grating. The beam will incident on the grating at the angle $\theta_{1}$ in medium with the refractive index $n_{1}$, accordingly. It can be calculated from the equation $n_{1}\sin\theta_{1}=\sin\theta .\;$

It should be noted that resonant wavelength of a limited beam, in particular a Gaussian beam, coincides with the resonant wavelength of the plane wave.

Amplitude distribution along the *x* coordinate on the grating surface is described with function a(x). It is practically equal to zero out of interval $\left[-x_{max}/2,\;x_{max}/2\right]$. Let us do Fourier transform of function $a\left(x\right)\exp\left(i2\pi u_{0}x\right)$, where $u_{0}=\frac{n_{1\;}}{\lambda }\sin\theta_{1},$ λ is wavelength of incident wave as follows:

(1) $$A\left(u\right)=\overset{\infty }{\underset{-\infty }{\int }}a\left(x\right)\exp\left(i2\pi u_{0}x\right)\exp\left(-i2\pi ux\right)dx$$

If equation $A_{0}\left(u\right)=\int_{-\infty }^{\infty }a\left(x\right)\exp\left(-i2\pi ux\right)dx$ is correct then it can be written:

(2) $$A\left(u\right)=A_{0}\left(u-u_{0}\right)$$

in correspondence to the sampling theorem [33], which is applied to Fourier transform.

Knowing A(u) it is possible to find $a\left(x\right)\exp\left(i2\pi u_{0}x\right)$ using the inverse Fourier transform as follows:

(3) $$a\left(x\right)\exp\left(i2\pi u_{0}x\right)=\int_{-\infty }^{\infty }A\left(u\right)\exp\left(i2\pi ux\right)du$$

Function A(u) is practically equal to zero out of interval $\left[u_{0}-u_{max}/2,\;u_{0}+u_{max}/2\right]$ and it is satisfied for Gaussian beam. The intervals should be such that constraints in the coordinate space and the frequency space do not lead to errors in the qualitative and quantitative results of the analysis. The spatial frequency interval $\left[u_{0}-u_{max}/2,\;u_{0}+u_{max}/2\right]$ is divided into *M* − 1 intervals, thus:

(4) $$u_{m}=u_{0}-\frac{M+1}{2}\delta u+m\delta u=\frac{n_{1}}{\lambda }\sin\theta_{m}$$

where $\delta u=\frac{u_{max}}{M-1}$, m is whole number which changes from 1 to M. Let selected that *M* is odd whole number. There will be M discrete samples of frequency u in this interval. According to the r sampling theorem [36], in order to pass from continuous coordinates and frequencies to discrete ones, and in order to use the discrete Fourier transform, the condition must be satisfied:

(5) $$x_{max}u_{max}=M-1\gg 1$$

Discrete coordinates can be expressed as follows:

(6) $$x_{m}=-\frac{x_{max}}{2}+\left(m-1\right)\frac{x_{max}}{M-1}=-\frac{x_{max}}{2}+\left(m-1\right)\delta x$$

The spatial frequency $u_{max}$ must satisfy the condition of Parseval equality [29]:

(7) $$n_{1}\cos\theta_{1}\overset{u_{0}+u_{max}/2}{\underset{u_{0}-u_{max}/2}{\int }}{\left|A\left(u\right)\right|}^{2}du\approx n_{1}{\cos\theta }_{1}\overset{\infty }{\underset{-\infty }{\int }}{\left|a\left(x\right)\right|}^{2}dx$$

Here, the “≈” sign, as is customary, means almost equal (asymptotically equal). It can be found $u_{max}$ based on this expression. The right-hand and left-hand sides of Equation (7) are proportional to the power of the incident beam on the grating. The following relation can be a criterion for choosing δu and M, taking into account that $\delta u=\frac{u_{max}}{M-1}$:

(8) $$n_{1}\cos\theta \overset{\frac{M-1}{2}}{\underset{j=-\frac{M-1}{2}}{\sum }}{\left|A\left(j\delta u\right)\right|}^{2}\delta u\approx n_{1}\cos\theta \overset{\infty }{\underset{-\infty }{\int }}{\left|a\left(x\right)\right|}^{2}dx$$

The value of the left-hand side of the ratio (8) follows to the value of the right-hand side with a decrease in δu, that is, with an increase in M. In addition, the follow relation must be satisfied [35]: 1δx≥umax, 1δu≥xmax. In our analysis, we will assume that 1δx=umax, 1δu=xmax.

Therefore, the beam with spatial distribution of field $a\left(x\right)\exp\left(i2\pi u_{0}x\right)$ can be represented by the sum of plane waves $A\left(u_{m}\right)\exp\left(i2\pi u_{m}x\right)\delta u$ as follows:

(9) $$a\left(x\right)\exp\left(i2\pi u_{0}x\right)\approx \overset{M}{\underset{m=1}{\sum }}A\left(u_{m}\right)\exp\left(i2\pi u_{m}x\right)\delta u$$

The result of diffraction of each plane wave $A\left(u_{m}\right)\exp\left(i2\pi u_{m}x\right)\delta u$ with number *m* can be calculated by the RCWA method. The amplitudes of the reflected and transmitted waves will be obtained and denoted by $r_{0m}$ and $t_{0m}$, respectively, for the zero order. In this case, for convenience, $r_{0m}$ and $t_{0m}$ are calculated at the unit amplitude of the incident wave. Thus, the total amplitudes of the reflected $r_{0}\left(x\right)$ and transmitted waves $t_{0}\left(x\right)$ at the homogeneous medium/grating interface can be obtained using the discrete inverse Fourier transform:

(10) $$r_{0}\left(x\right)=\overset{M}{\underset{m=1}{\sum }}A\left(u_{m}\right)r_{0m}\left(u_{m}\right)\exp\left(i2\pi u_{m}x\right)\delta u$$

(11) $$t_{0}\left(x\right)=\overset{M}{\underset{m=1}{\sum }}A\left(u_{m}\right)t_{0m}\left(u_{m}\right)\exp\left(i2\pi u_{m}x\right)\delta u$$

Using Equations (10) and (11), it is possible to calculate values proportional to the power distributions of the reflected wave along the grating and the wave passing through the grating in accordance with the expressions:

(12) $$R_{0}\left(x\right)={\left|r_{0}\left(x\right)\right|}^{2}n_{1}\cos\theta_{1}$$

(13) $$T_{0}\left(x\right)={\left|t_{0}\left(x\right)\right|}^{2}n_{3}\cos\theta_{3}$$

If the grating medium and homogeneous media adjacent to the grating are not absorbing, then with the correct choice of *M*, $u_{max},\;\;x_{max}$ the follow condition must be satisfied:

(14) $$n_{1}\cos\theta_{1}\overset{\infty }{\underset{-\infty }{\int }}{\left|a\left(x\right)\right|}^{2}dx\approx \int_{-x_{max}/2}^{x_{max}/2}\left[R_{0}\left(x\right)+T_{0}\left(x\right)\right]dx\approx n_{1}\cos\theta_{1}\overset{u_{0}+u_{max}/2}{\underset{u_{0}-u_{max}/2}{\int }}{\left|A\left(u\right)\right|}^{2}du$$

The less the integrals differ from each other, the more accurate the analysis is. We express the relative powers of the grating transmission and the reflection from the grating as follows:

(15) $$P_{r}=\frac{\int_{-x_{max}/2}^{x_{max}/2}R_{0}\left(x\right)dx}{n_{1}\cos\theta_{1}\int_{-\infty }^{\infty }{\left|a\left(x\right)\right|}^{2}dx},\;P_{t}=\frac{\int_{-x_{max}/2}^{x_{max}/2}T_{0}\left(x\right)dx}{n_{1}\cos\theta_{1}\int_{-\infty }^{\infty }{\left|a\left(x\right)\right|}^{2}dx}$$

Obviously, the condition $P_{r}$ + $P_{t}\approx$ 1 must be satisfied with such definition of $P_{r}$ and $P_{t}$ in the absence of absorption in the structure. It corresponds to the energy conservation law. Therefore, it can be concluded that results of the analysis by the proposed approach correspond to the energy conservation law for phase gratings.

The integrals in the numeral Equation (15) can be replaced by summation to increase the speed of numerical calculation as follows:

(16) $$\int_{-x_{max}/2}^{x_{max}/2}R_{0}\left(x\right)dx\approx \overset{\frac{M-1}{2}}{\underset{j=-\frac{M-1}{2}}{\sum }}R_{0}\left(j\delta x\right)\delta x,\;\int_{-x_{max}/2}^{x_{max}/2}T_{0}\left(x\right)dx\approx \overset{\frac{M-1}{2}}{\underset{j=-\frac{M-1}{2}}{\sum }}T_{0}\left(j\delta x\right)\delta x\;\;\;$$

## 3. Numerical Analysis of the Gaussian Beam Diffraction by the Infinite Grating

Let consider the diffraction of the Gaussian beam by the infinite grating. The Gaussian beam and grating are shown in Figure 1. The Gaussian beam can be described by the following equation in the coordinate system $\left(x_{1},\;z_{1}\right)$:

(17) $$E\left(x_{1},z_{1}\right)=\exp\left[-\pi {\left(\frac{x_{1}}{L}\right)}^{2}\right]\exp\left(i\frac{2\pi n_{1}}{\lambda }z_{1}\right)$$

where the first factor is the amplitude of this beam $a\left(x_{1}\right)=\exp\left[-\pi {\left(\frac{x_{1}}{L}\right)}^{2}\right].$

In the coordinate system (x,z) the corresponding distribution of the Gaussian beam field will have the form:

(18) $$E\left(x,z\right)=\exp\left[-\pi {\left(\frac{x\cos\theta_{1}}{L}\right)}^{2}\right]\exp\left[i2\pi \left(\frac{n_{1}}{\lambda }\sin\theta_{1}\;x+\frac{n_{1}}{\lambda }\cos\theta_{1}z\right)\right].$$

Thus, it can be written:

(19) $$a\left(x\right)=\exp\left[-\pi {\left(\frac{x\cos\theta_{1}}{L}\right)}^{2}\right]\exp\left(i2\pi \frac{n_{1}}{\lambda }\sin\theta_{1}x\right)=\exp\left[-\pi {\left(\frac{x\cos\theta_{1}}{L}\right)}^{2}\right]\exp\left(i2\pi u_{0}x\right).$$

The following grating parameters $n_{1}=1.5$ is the refractive index of the tested medium, $n_{20}=1.525$ is the average refractive index of the grating medium, $n_{3}=1.38$ is the refractive index of the substrate, and d=1.86860 μm is the grating thickness have been used in our numerical studies. The resonant wavelength at the plane wave incidence, at which the reflection coefficient from the structure is equal to unity, was 1.064 μm. This wavelength was selected in terms of increasing sensitivity with increasing wavelength [13]. The angle of incidence of the beam on the grating in air θ is 20 angular degrees under resonance. Several amplitudes of modulation of the refractive index of the grating medium $n_{m}=0.017,\;0.025,\;0.035,\;0.05$ have been analyzed. The change in $n_{m}$ will lead to the change in the grating period to achieve resonance since the resonant wavelength is 1.064 μm for all four cases. Table 1 shows the grating resonance periods. The resonance parameters of the grating at the different wavelength with constant refractive indices and the beam resonant angle of incidence on the grating can be calculated based on the results of the analysis at the wavelength of 1064 nm, grating period Λ and grating thickness d. The resonance period and thickness are $\Lambda_{\lambda }=\frac{\lambda }{1064}\Lambda ,$ $d_{\lambda }=\frac{\lambda }{1064}d$ for the wavelength λ. The wavelength of 1064 nm was chosen from the point of view of the mastered production of cheap YAG^Nd^3+^ lasers pumped by the semiconductor laser.

As follows from the Table 1, the grating period changes in the fifth significant digit when the modulation amplitude changes from 0.017 to 0.05.

In computer analysis, $u_{max}$ was chosen from the condition:

(20) $$L\exp\left[-\pi {\left(L\frac{u_{max}}{2}\right)}^{2}\right]=10^{-7}$$

The grating period changes in the fifth significant digit when the modulation amplitude changes from 0.017 to 0.05 as follows from the Table 1.

**Figure 2:**
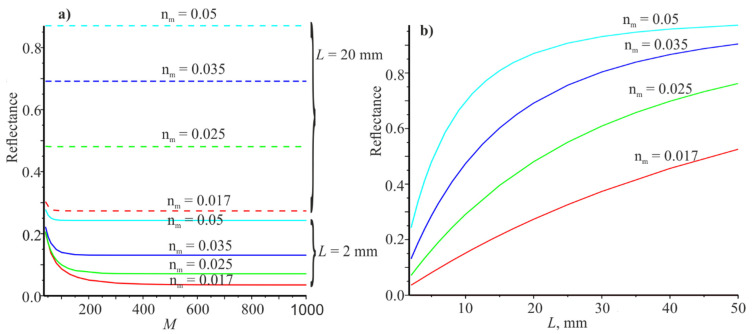
Dependences of the reflection coefficients on M for L=2 mm (4 lower curves for) end L=20 mm (4 upper curves) (**a**). The dependence of the reflection coefficients on L at M=501 (**b**). The red, green, blue, and cyan curves correspond to the modulus coefficients of the refractive index $n_{m}:0.017,\;0.025,\;0.035,\;0.05$.

If L=15,000 μm, then $u_{max}=0.00095\;{\mu m}^{-1}$. It is possible to ensure high accuracy of numerical calculations under this condition. Obviously, it first need to calculate the dependences of the reflection and transmission coefficients on M for two values of L. The corresponding dependences are shown in Figure 2a. The dependences of the reflection and transmission coefficients on L for M=501 are shown in Figure 2b. It should be noted that $P_{r}$ + $P_{t}=$ 0.9999999903 when $u_{max}$ determined according to the Equation (20). That is, the calculations can be performed with high accuracy for the Gaussian beam with this choice of $u_{max}$.

Analyzing Figure 2a, it can be seen that the most sensitive reflection coefficient (P) in terms of power in M is for $n_{m}=0.017$ at L=2 mm. $P_{r}$ changes from 0.036094 to 0.03573 when M changes from 501 to 1001, that is, insignificantly. Therefore, all subsequent calculations were carried out at M=501. Figure 2b shows the dependences of $P_{r}$ on the half-width of the beam L. It can be seen that the reflection coefficients increase monotonically with increasing L. Thus, it can be assumed that $P_{r}$ tends to unity with increasing L. It should be noted that the sum $P_{r}$ + $P_{t}=1$, since we used $u_{max}$ large enough according to Equation (20).

**Figure 3:**
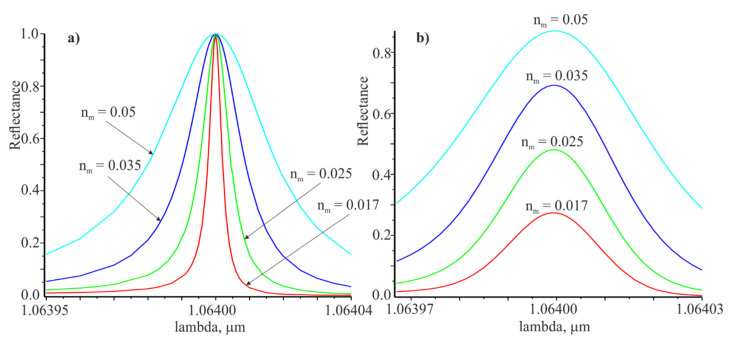
Spectral dependences of the reflection coefficient for the plane wave (**a**) and the Gaussian beam L=20 at the resonance angle θ=π/9 (**b**). The red, green, blue and cyan curves correspond to the modulation coefficients of the refractive index $n_{m}:0.017,\;0.025,\;0.035,\;0.05$.

The following figure shows the spectral dependences when the plane wave (Figure 3a) and the Gaussian beam at L=20 mm (Figure 3b) are incident on the grating The resonance wavelengths for both cases coincide, but the half-widths of the resonance curves are large for the Gaussian beam compared to the plane wave.

**Figure 4:**
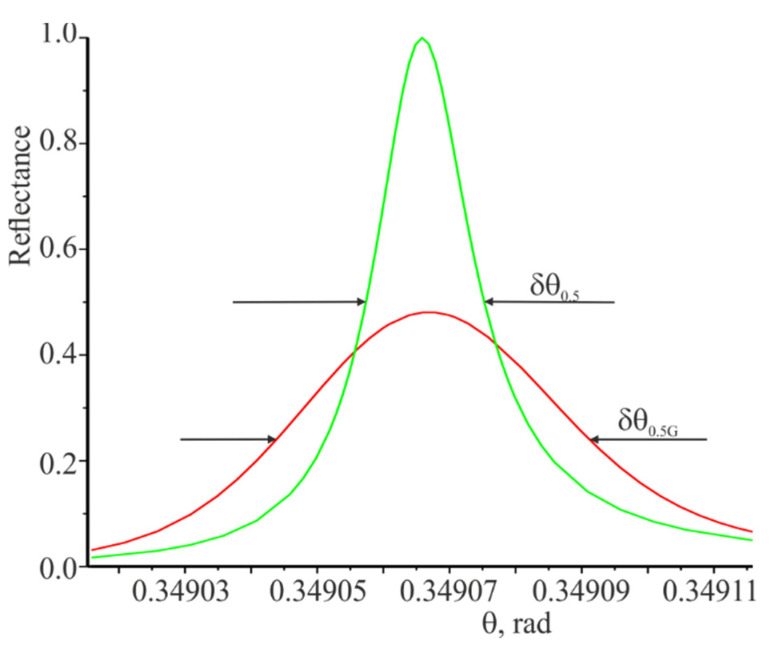
Angular dependences of the reflection coefficient at the incidence of the plane wave and the Gaussian beam on the grating. The green curve is the incidence of the plane wave on the grating. The red curve is the incidence of the Gaussian beam at $L=20\;\mathrm{mm},\;n_{m}=0.025$. The resonant wavelength is 1.064 μm.

The angular dependences of the reflection coefficient at the incidence of the plane wave and the Gaussian beam on the grating are presented in Figure 4. There are indicated the widths of the resonance curves ${\delta \theta }_{0.5}$ and ${\delta \theta }_{0.5G}$ at the level of half of the corresponding maximum values. In the same way, the widths of the spectral resonance curves presented in Figure 3 were determined.

The spectral and angular widths of the resonant response for the Gaussian beam are wider in comparison to the width of the resonance responses for the plane wave. Apparently, is true not only for the limited Gaussian beam. The reflection coefficient from the grating is equal to unity at the resonant wavelength when the plane wave is incident. However, the reflection coefficient for the limited beam is less than unity and is directed to unity with increasing beam width and modulation amplitude of the refractive index $n_{m}$.

**Figure 5:**
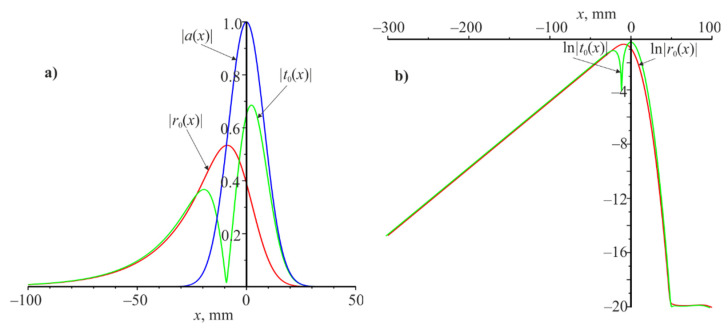
Amplitude modulus distribution $\left|a\left(x\right)\right|,\left|r_{0}\left(x\right)\right|$ and $\left|t_{0}\left(x\right)\right|$ along the x coordinate (**a**) and distribution $\ln\left|r_{0}\left(x\right)\right|$ and $\ln\left|t_{0}\left(x\right)\right|$ along the x coordinate (**b**). Resonant wavelength is 1.064 μm and resonant angle is $\theta =20^{o}$ at the L=20 mm.

High-information curves are dependencies $\left|r_{0}\left(x\right)\right|,$ $\left|t_{0}\left(x\right)\right|$ and especially $\ln\left[\left|r_{0}\left(x\right)\right|\right]$ and $\ln\left[\left|t_{0}\left(x\right)\right|\right]$, which are shown in Figure 5. These curves were calculated under resonance for the Gaussian beam ($L=20\;\mathrm{mm},\;n_{m}=0.025$). It can be seen that the reflected beam (red) is much wider than the incident beam (blue) and expands to the left along the x coordinate for a considerable distance (see Figure 5). The transmitted beam (green) is split into two parts and also expands to the left over a considerable distance. The reflected and transmitted beams are shifted to the left relative to the incident beam.

Analyzing Figure 5a, we can assume that the modules $\left|r_{0}\left(x\right)\right|$ and $\left|t_{0}\left(x\right)\right|$ decreases exponentially as x decreases from −30 to −100 mm. This assumption is confirmed by Figure 5b, where is linear dependence of $\ln\left|r_{0}\left(x\right)\right|$ and $\ln\left|t_{0}\left(x\right)\right|$. It is possible to determine the slope coefficient of these straight lines for different $n_{m},$ which we denote by $\alpha_{rad}$ on these linear dependences. These $\alpha_{\mathrm{rad}}$ values are shown in Table 2 (fifth row) for four values of $n_{m}$ (first row). It should be noted that $\alpha_{\mathrm{rad}}$ does not depend on the Gaussian beam width but depends on $n_{m}$. These figures show that waveguide mode is excited under resonance. It propagates from right to left and it decays exponentially during propagation due to interaction with the grating. The energy losses of the waveguide mode go to the reflections and transmissions of the grating. Due to which there is a spatial expansion of the reflected and transmitted beams.

**Figure 6:**
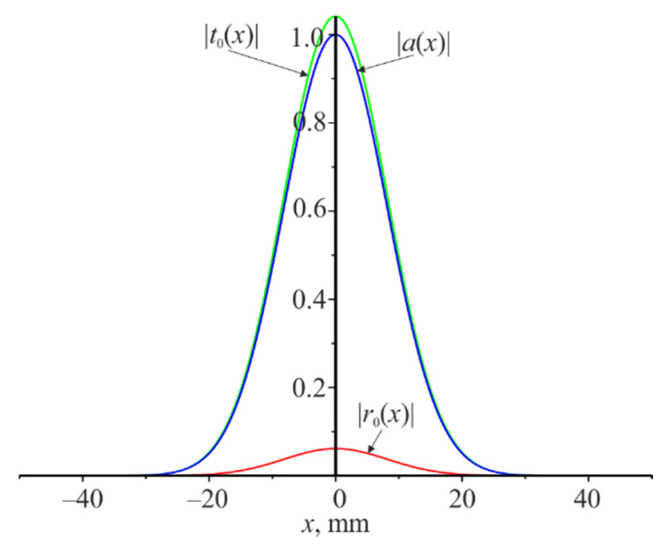
Amplitudes modules distribution of |a(x)| (blue), $\left|r_{0}\left(x\right)\right|\;$(red), and $\left|t_{0}\left(x\right)\right|$ (green) along the x coordinate. The wavelength is 1000 nm, the width of the Gaussian beam incident on the grating is L=20 mm.

The behavior of the spatial distribution of the amplitudes of the reflected and transmitted beams in the absence of resonance will be quite different. The corresponding dependences for the wavelength of 1000 nm are shown in Figure 6.

Therefore, the distributions of the field amplitude of the reflected and transmitted beams differ significantly in shape from the distribution of the amplitude of the incident beam on the grating under resonance. All three beams are similar in shape under the absence of resonance.

It is quite far from the resonance wavelength of 1064 nm. It can be seen that there is no expansion of the reflected and transmitted beams and no change in their shape. There is the insignificant amplitude $P_{r}=0.0038$ of the reflected beam. The same will be the reflection coefficient when the plane wave is incident on the grating.

Rows 3 and 4 of the Table 2 show the reflection coefficients from the grating under resonance at L=2 mm and L=20 mm for four values of $n_{m}$, respectively. The sixth row of the Table 2 shows the widths of the spectral resonances $\delta \lambda_{0.5}$ when the plane wave is incident on the grating. They are determined based on the curves shown in Figure 3a. The seventh row of the Table 2 contains ${\delta \lambda }_{0.5\alpha }$, calculated by the following analytical expression [10,30]

(21) $${\delta \lambda }_{0.5\alpha }=\alpha_{rad}\lambda \Lambda /\pi$$

There are a good agreement between the data given in rows 6 and 7. It confirms the correctness of the proposed method for calculating the limited beam diffraction by the diffraction grating. Row 8 shows the spectral widths of the resonance curves $\delta \lambda_{0.5G}$ when the Gaussian beam is incident on the grating. Analyzing the data of lines 6 … 8, we can conclude that the widths of the resonance curves grow with increasing $n_{m}$.

Row 9 shows the widths of angular resonances at the plane wave the incidence on the grating obtained based on the curves partially shown in Figure 4. The data in line 10 is determined by the following equation [10,30]:

(22) $${\delta \theta }_{0.5\alpha }=\alpha_{rad}\lambda /\left(\pi \cos\theta \right)$$

It can be seen a good coincidence of data rows 9 and 10. Row 11 shows the widths of the angular resonance curves for the Gaussian beam.

Therefore, the attenuation index of the waveguide mode during propagation determines the widths of the resonances of the spectral and angular responses when a plane wave is incident on the grating (see Equations (21) and (22)).

Rows 12 and 13 show the spectral sensitivities $S_{\lambda }$ and $S_{\lambda G}$ to the change in the refractive index $n_{1}$ of the tested medium when the plane wave and the Gaussian beam are incident on the grating, respectively. Spectral sensitivities were determined as follows:

(23) $$S_{\lambda }=\frac{\lambda_{rez}\left(n_{1}+\Delta n_{1}\right)-\lambda_{rez}\left(n_{1}-\Delta n_{1}\right)}{2\Delta }.\;$$

There is $\Delta n_{1}=0.0001$ in our numerical calculations. Similarly, the sensitivity was determined for the Gaussian beam. The resonant wavelength is 1.064 μm t the resonance angle $\theta_{rez}=20^{{}^{\circ}}$ for $n_{1}=1.5$. The new resonance wavelengths $\lambda_{rez}\left(n_{1}\pm \Delta n_{1}\right)$ were searched after changing $n_{1}$ by $n_{1}\pm \Delta n_{1}$. These data were substituted into Equation (23). Comparing the data in rows 12 and 13 of Table 2, we see that the sensitivities for both cases are the same and depend very little on $n_{m}$.

Rows 14 and 15 show the angular sensitivity $S_{\theta }$ and $S_{\theta G}$ to the change in the refractive index $n_{1}$ of the tested medium when the plane wave and the Gaussian beam are incident on the grating. The angular sensitivities were determined as follows:

(24) $$S_{\theta }=\frac{\theta_{rez}\left(n_{1}+\Delta n_{1}\right)-\theta_{rez}\left(n_{1}-\Delta n_{1}\right)}{2\Delta n_{1}}$$

The sensitivity of the Gaussian beam was determined in the similar way.

It was found that the spectral and angular sensitivity to the change in the refractive index n_1 are the same for the plane wave and the Gaussian beam and increase slightly with increasing of $n_{m}$. The fact that the sensitivities (angular and spectral) are the same when the plane wave and the Gaussian beam are incident on the grating can be explained by the fact that plane waves propagate in a very narrow range of angles in the Gaussian beam expansion. They are determined by the interval of spatial frequencies $\left[u_{0}-u_{max}/2,\;u_{0}+u_{max}/2\right]$. We also see that the sensitivity very little depends on the modulation amplitude of the refractive index $n_{m}$. It is much less than the average refractive index of the grating medium $n_{20}$.

The structure under study is essentially a planar waveguide and the following condition must be satisfied under resonance [10,13,17]:

(25) $$\frac{2\pi }{\lambda }\sin\theta -\frac{2\pi }{\Lambda }+\beta \approx 0$$

where β is propagation constant of the planar waveguide. The resonance grating period changes in the fifth significant digits (second row of Table 2) when $n_{m}$ changes from 0.017 to 0.05 (the first row of the table). Therefore, propagation constant will be insignificantly changed at change of $n_{m}$. As a result, there will be little change in spectral and sensitiveness on the change of $n_{m}.$

Rows 16 and 17 of Table 2 show the ratios $S_{\lambda }/{\delta \lambda }_{0.5}$ and $S_{\theta }/{\delta \theta }_{0.5}$ for the plane wave, which are calculated based on data from rows 6, 9, 12, and 14. It can be seen that:

(26) $$\frac{S_{\lambda }}{{\delta \lambda }_{0.5}}\sim \frac{S_{\theta }}{{\delta \theta }_{0.5}}$$

The last equation was obtained in [10] based on Equation (25). Rows 18 and 19 of Table 2 show similar equations for the incidence of the Gaussian beam on the grating using data in rows 8, 11, 13 and 15. We also see a good coincidence of the results of rows 18 and 19 for all $n_{m}$. Thus, it can be written:

(27) $$\frac{S_{\lambda G}}{{\delta \lambda }_{0.5G}}\sim \frac{S_{\theta G}}{{\delta \theta }_{0.5G}}$$

Thus, the advantage of proposed approach is in analysis of the cases that are closest to practice. The diffraction of a Gaussian beam with a width of two mm or more have been studied. Numerical simulation of the Gaussian beam diffraction by the grating using the finite element method showed that analysis took a very long time (the beam width was 1–2 mm) and the calculation results were not credible. Finite element methods, finite-difference frequency-domain methods require computer mesh with a small step. Therefore, it is convenient to use these methods for diffraction of a plane wave by the grating, where the calculation is performed within one period. They can also be used to analyze the diffraction of the beam with very small width (several periods). In [22], diffraction of the limited beam size from 3 to 20 periods was studied using the finite-difference frequency-domain method. In this work, the diffraction of limited beams was studied in width, that is, with a width of several periods. The number of grid points per wavelength was 20 (see [22]). Possibility to calculate diffraction of the beam by grating with the beam size of 1 mm or more is the main advantage of our method. It has a practical importance. Our method, after some modification, can be used for very narrow beams. However, in this case, perhaps tens of thousands of plane waves must have to use. It leads to a significant increase in computer calculations. It can be argued that the proposed method and the finite-difference frequency-domain method and other methods using a mesh complement each other at the analysis of finite-width beams diffraction by the diffraction gratings.

## 4. Conclusions

The limited beam diffraction by the phase grating has been studied by proposed method of analysis. It is found that waveguide mode is excited under resonance and decays exponentially during propagation. The spatial distribution of the amplitude of the limited beam is described with the Gaussian function. The analysis was carried out near the resonant wavelength for four values of the modulation amplitude of the grating medium refractive index. It was found that the resonant wavelength is equal to the resonant wavelength when the plane wave is incident on the grating. In case when the Gaussian beam is incident on the grating. The spectral and angular widths of the resonant response for the Gaussian beam are wider in comparison with the width of the resonant responses for the plane wave. It is apparently true not only for the limited Gaussian beam. The reflection coefficient from the grating is equal to unity at the resonant wavelength in case of plane wave incidence. However, the reflection coefficient for the limited beam is less than unity and is directed towards unity with the increase in the beam width and modulation amplitude of the refractive index $n_{m}$.

The proposed method for analyzing diffraction of the limited Gaussian beam on the phase grating corresponds to the energy conservation law. The sum of the powers of the reflected and transmitted beams is equal to the power of the incident beam.

It was found that the amplitudes spatial distribution of the reflected and transmitted beams in the zero diffraction order differs in shape from the distribution of the amplitude of the incident beam under resonance. The widths of the reflected and transmitted beams are higher than the width of the incident beam. If there is no resonance, then the spatial distribution of the amplitudes of the incident, reflected and transmitted beams are similar in the shape. It was shown that the waveguide mode is excited under resonance. The amplitude of which decreases exponentially. The radiation loss index $\alpha_{\mathrm{rad}}$ determines the width of the resonant spectral and angular losses at the FWHM for the plane wave in accordance with Equations (21) and (22). The reflected and transmitted beams expand spatially due to the interaction of the waveguide mode with the grating under resonance.

It was found that the spectral and angular sensitivity to the change in the refractive index $n_{3}$ are the same for the plane wave and the Gaussian beam and increase slightly with increasing of $n_{m}$. Numerical calculations show that $\frac{S_{\lambda }}{{\delta \lambda }_{0.5}}\sim \frac{S_{\theta }}{{\delta \theta }_{0.5}}>$ $\frac{S_{\lambda G}}{{\delta \lambda }_{0.5G}}\sim \frac{S_{\theta G}}{{\delta \theta }_{0.5G}}$. It is consistent with the results of [10,13].

Generally, it can be concluded that results of this work will be useful for the design of refractive index sensors of biological solutions based on water or alcohol. The principle of operation of sensors is based on waveguide resonance in dielectric gratings and sensitivity of the resonance based sensors significantly exceeds the sensitivity of holographic sensors. It should be noted that actual waveguide resonance based volume phase gratings can be fabricated by the holographic recording using the symmetric two-beam setup for the fabrication of transmission gratings. The detailed description of manufacturing process is presented in [10]. The high uniformity in thickness and accurate reproduction of the periodicity allow us to expect a good fitting between the results of numerical modeling and experimental data.

## Figures and Tables

**Table 1:** Resonant grating periods for 4 values of *n_m_* cited.

$n_{m}$	0.017	0.025	0.035	0.05
Λ, nm	573.518	573.527	573.544	573.580

**Table 2:** Calculation results of grating properties under waveguide resonance at 4 values of *n_m_* for plane wave and Gaussian beam.

Parameter	No	Number Data of Parameters				Calculation Conditions
Modulation $n_{m}$	1	0.017	0.025	0.035	0.050	$n_{2}\left(x\right)=n_{20}+n_{m}\cos\left(\frac{2\pi }{\Lambda }x\right)$
Lambda Λ, nm	2	573.5418	573.5273	573.5441	573.5797	Grating period
$P_{r}$	3	0.0351	0.0710	0.1310	0.2429	L=2 mm
$P_{r}$	4	0.2735	0.4804	0.6917	0.8703	L=20 mm
$\alpha_{\mathrm{rad}},{\;\mathrm{mm}}^{-1}$	5	0.02286	0.04943	0.09686	0. 1976	L=2…20 mm
${\delta \lambda }_{0.5}$, nm	6	4.43 × 10^−3^	9.58 × 10^−3^	18.75 × 10^−3^	38.26 × 10^−3^	Plane wave
${\delta \lambda }_{0.5\alpha }$, nm	7	4.44 × 10^−3^	9.60 × 10^−3^	18.82 × 10^−3^	38.38 × 10^−3^	${\delta \lambda }_{0.5\alpha }=\alpha_{rad}\lambda \Lambda /\pi$
${\delta \lambda }_{0.5G},\;\mathrm{nm}$	8	22.22 × 10^−3^	25.28 × 10^−3^	31.52 × 10^−3^	46.98 × 10^−3^	L=20 mm Gaussian beam
${\delta \theta }_{0.5}$, mrad	9	0.00825	0.0179	0.0351	0.0715	Plane wave
${\delta \theta }_{0.5\alpha }$, mrad	10	0.00824	0.0179	0.0349	0.0712	${\delta \theta }_{0.5\alpha }=\alpha_{rad}\lambda /\left(\pi \cos\theta \right)$
${\delta \theta }_{0.5G}$, mrad	11	0.0413	0.0473	0.0590	0.0878	L=20 mm Gaussian beam
$S_{\lambda },\;\mathrm{nm}$	12	75.9	76.0	76.2	76.75	Plane wave
$S_{\lambda G},\;\mathrm{nm}$	13	75.9	76.0	76.2	76.75	L=20 mm Gaussian beam
$S_{\theta },$ mrad	14	141.2	142.4	142.6	143.4	Plane wave
$S_{\theta G},$ mrad	15	141.2	142.4	142.6	143.4	L=20 mm Gaussian beam
$S_{\lambda }/{\delta \lambda }_{0.5}$	16	17,133	7933	4064	2006	Plane wave
$S_{\theta }/{\delta \theta }_{0.5}$	17	17,115	7955	4063	2006	Plane wave
$S_{\lambda G}/{\delta \lambda }_{0.5G}$	18	3416	3006	2418	1634	L=20 mm Gaussian beam
$S_{\theta G}/{\delta \theta }_{0.5G}$	19	3419	3011	2417	1633	L=20 mm Gaussian beam

## Data Availability

Data sharing not applicable.
